# RSPO2-induced ferroptosis via PTBP1-mediated FSP1 mRNA decay suppresses breast cancer progression

**DOI:** 10.3389/fonc.2026.1813451

**Published:** 2026-06-09

**Authors:** Shuyun Jiang, Hongwei Ma, Zhanwei Du, Zhijun Ma, Xiaowu Wang

**Affiliations:** 1 College of Clinical Medicine, Qinghai University, Xining, Qinghai, China; 2 Department of Surgical Oncology, The Affiliated Hospital of Qinghai University, Xining, Qinghai, China

**Keywords:** RSPO2, breast cancer, ferroptosis, FSP1, PTBP1

## Abstract

**Introduction:**

Breast cancer is the most common malignant tumor worldwide, and its high heterogeneity limits the efficacy of current clinical therapies. R-spondin 2 (RSPO2) expression is closely associated with breast cancer subtypes, but the precise mechanisms by which it drives disease progression, particularly its relationship with ferroptosis, remain unclear.

**Methods:**

RSPO2 expression was examined in primary breast cancer specimens and in MCF-7 and MDA-MB-231 cell lines. Functional assays, including proliferation, migration, and invasion tests, were performed following RSPO2 overexpression. *In vivo* tumor growth was assessed using a xenograft mouse model. Mechanistically, immunoprecipitation, ubiquitination assays, RNA sequencing (RNA-seq), RIP-qPCR, mRNA stability assays, and ferroptosis-related measurements (ROS, GSH, MDA, Fe^2+^) were employed to identify interacting proteins and downstream targets. Rescue experiments with FSP1 overexpression were conducted to validate pathway specificity.

**Results:**

RSPO2 was consistently downregulated in malignant breast cancer tissues. Restoring RSPO2 expression markedly suppressed cell proliferation, migration, and invasion in vitro and inhibited tumor growth in vivo. Mechanistically, RSPO2 recruited the E3 ubiquitin ligase TRIM21 to promote PTBP1 ubiquitination and proteasomal degradation. RNA-seq and validation assays revealed that PTBP1 bound the 3′UTR of ferroptosis suppressor protein 1 (FSP1) mRNA, enhancing its stability. RSPO2 overexpression reduced PTBP1 levels, leading to decreased FSP1 mRNA stability and expression. Consequently, RSPO2 overexpression triggered ferroptosis, as evidenced by ROS accumulation, GSH depletion, and elevated MDA and Fe^2+^ levels. These effects were effectively reversed by FSP1 overexpression, which also abrogated the RSPO2 induced inhibition of cell proliferation, migration, and invasion.

**Discussion:**

This study demonstrates that RSPO2 functions as a tumor suppressor in breast cancer by recruiting TRIM21 to degrade PTBP1, thereby reducing FSP1 mRNA stability and promoting ferroptosis. The RSPO2/PTBP1/FSP1 signaling axis is identified for the first time in breast cancer and represents a promising novel therapeutic target.

## 1 Introduction

Breast cancer is the most frequently diagnosed malignancy among women worldwide, and its pronounced heterogeneity remains a central challenge in clinical management (1). Based on gene expression profiling, breast cancers are classified into luminal A, luminal B, human epidermal growth factor receptor 2 (HER2)–enriched, and triple-negative breast cancer (TNBC) subtypes. These groups differ markedly in their driver mutations, proliferative capacity, metastatic potential, and treatment responsiveness; in particular, TNBC—devoid of established therapeutic targets—carries the poorest prognosis (2). Although the combined use of surgery, radiotherapy, chemotherapy, and targeted therapies has significantly improved overall survival, therapeutic resistance, tumor recurrence, and distant metastasis remain unresolved obstacles (3). Consequently, uncovering the deep molecular mechanisms that drive subtype-specific progression in breast cancer is imperative for advancing precision oncology.

R-spondin 2 (RSPO2), a key member of the R-spondin family, acts as an activator of Wnt/β-catenin signaling by binding to LRP5/6 and LGR4/5/6 receptors, thereby stabilizing the Wnt receptor complex and playing a central role in embryonic development, stem cell maintenance, and tissue regeneration (4). However, the function of RSPO2 in tumorigenesis and progression displays marked tissue specificity and context dependency. Pan-cancer analysis revealed that, compared with normal tissues, RSPO2 expression is significantly downregulated in 11 tumor types and upregulated in five others (5). In colorectal cancer, RSPO2 gene translocations or fusions produce gain-of-function variants, identifying RSPO2 as a critical oncogenic driver (6). Conversely, in hepatocellular carcinoma, RSPO2 acts as a tumor suppressor, and its decreased expression correlates with poorer patient prognosis (7). Notably, in breast cancer the role of RSPO2 appears to be both complex and incompletely defined. Emerging data indicate that RSPO2 expression and function vary across molecular subtypes: high RSPO2 levels are positively associated with basal‐like and HER2‐enriched tumors, yet inversely correlated with luminal A and luminal B cancers, suggesting subtype‐specific contributions to tumorigenesis and progression (6). The molecular mechanisms underlying these divergent effects remain to be elucidated. In this study, we perform a comprehensive analysis of RSPO2 expression across breast cancer subtypes, investigate its impact on Wnt/β-catenin signaling activity and cellular phenotypes in subtype‐defined models, and assess its potential as a prognostic biomarker and therapeutic target. However, the precise role and molecular mechanisms by which RSPO2 contributes to breast cancer initiation and progression remain poorly defined. Polypyrimidine Tract-Binding Protein 1 (PTBP1) is a pivotal RNA-binding protein that governs multiple post-transcriptional processes of its target mRNAs—including alternative splicing, stability, subcellular transport and translation—and thereby orchestrates key cellular programs such as differentiation, metabolic reprogramming and stress responses (8). PTBP1 is aberrantly overexpressed in a variety of malignancies—including breast cancer, glioma and colorectal carcinoma—and drives tumor cell proliferation, invasion, metastasis and therapy resistance by activating the PI3K/Akt pathway, enhancing glycolysis, inhibiting apoptosis and promoting epithelial–mesenchymal transition (EMT) (9) (PMID: 40579921). For example, in breast cancer cells PTBP1 facilitates growth through PTEN/Akt signaling and induction of autophagy (10). Moreover, knockdown of the deubiquitinase USP46 increases K48-linked ubiquitination and proteasomal degradation of PTBP1, thereby reducing the PKM2/PKM1 ratio, decreasing glycolytic flux and enhancing tamoxifen sensitivity (11). Nevertheless, the upstream regulatory network controlling PTBP1 remains poorly characterized, and the mechanisms by which extracellular cues—such as the secreted protein RSPO2—modulate PTBP1 expression and activity have yet to be elucidated.

In recent years, ferroptosis—an iron‐dependent form of regulated cell death triggered by the excessive accumulation of lipid peroxides—has been recognized as morphologically, biochemically and genetically distinct from apoptosis, necrosis and autophagy (12). Cellular defense against ferroptosis relies on two principal protective systems. The first is the classical glutathione (GSH)/glutathione peroxidase 4 (GPX4) axis, in which GPX4 consumes GSH to reduce lipid hydroperoxides (PLOOHs) to non-toxic lipid alcohols (PLOHs), thereby directly halting the lipid peroxidation chain reaction. The second is an essential, GPX4–GSH-independent parallel pathway mediated by ferroptosis suppressor protein 1 (FSP1) (13). Furthermore, FSP1 employs an NADH‐dependent mechanism to reduce ubiquinone (CoQ10) to its active antioxidant form, ubiquinol. Ubiquinol can directly scavenge lipid radicals or regenerate vitamin E, thereby arresting lipid peroxidation and conferring resistance to ferroptotic cell death (14). Collectively, the GSH/GPX4 axis and the FSP1–CoQ10 pathway form the two central defense lines against ferroptosis, which are crucial for preserving cellular redox homeostasis and modulating the onset and progression of various diseases. Ferroptosis suppressor protein 1 (FSP1), originally identified as a p53‐responsive gene and thus also referred to as PRG3 (p53‐responsive gene 3), is markedly overexpressed in a variety of malignancies—including lung adenocarcinoma, breast cancer and pancreatic cancer—and its elevated expression correlates with poor prognosis and chemoresistance (15). For instance, in hepatocellular carcinoma, FSP1 activates the SIRT1/PGC-1α signaling axis to drive mitochondrial biogenesis, thereby augmenting tumor cell metabolic activity and invasiveness. Conversely, FSP1 knockdown markedly impairs hepatocellular carcinoma cell migration and reduces the formation of pulmonary metastatic nodules (16). Likewise, elevated FSP1 expression in breast cancer correlates with poorer clinical outcomes—namely reduced overall survival and increased risk of metastasis—and pharmacological inhibition of its oxidoreductase activity effectively delays disease progression (17). However, to date no studies have examined whether—and how—the secreted ligand RSPO2 regulates FSP1 expression or function, leaving this potential axis unexplored.

This study aims to elucidate the molecular mechanism by which R-spondin 2 (RSPO2) induces ferroptosis in breast cancer cells through suppression of the PTBP1/FSP1 axis, thereby impairing malignant progression. By defining this regulatory cascade, we seek to establish a new theoretical framework and identify potential therapeutic targets for RSPO2-based targeted interventions in breast cancer.

## 2 Materials and methods

### 2.1 Cell culture

MCF-10A cells were maintained in DMEM/F-12 medium (Procell, PM150312) supplemented with 5% horse serum (Procell, 164215), 20 ng/mL epidermal growth factor (Sigma-Aldrich, E9644), 0.5 μg/mL hydrocortisone (Sigma-Aldrich, H0888), 100 ng/mL cholera toxin (Sigma-Aldrich, C8052), 10 μg/mL insulin (Procell, PB180432) and 1% penicillin–streptomycin solution (Procell, PB180120). T47D cells were cultured in RPMI-1640 medium (Procell, PM150110) containing 10% fetal bovine serum (Gibco, A5669701) and 1% penicillin–streptomycin solution (Procell, PB180120). MCF-7, MDA-MB-231 and BT-549 cells were maintained in DMEM (Procell, PM150210) supplemented with 10% fetal bovine serum and 1% penicillin–streptomycin solution. All cell lines were incubated at 37 °C in a humidified atmosphere with 5% CO_2_.

### 2.2 Reverse transcription–quantitative PCR

Total RNA was extracted from cultured cells or tissue samples using TRIzol Reagent (Thermo Fisher Scientific, 15596018CN) according to the manufacturer’s protocol. The human breast cancer and adjacent normal tissue samples used in this study were obtained from the Department of Breast and Thyroid Tumor Surgery, Affiliated Hospital of Qinghai University, with written informed consent from all patients. The experimental protocol was approved by the Ethics Committee of the Affiliated Hospital of Qinghai University. One microgram of RNA was reverse‐transcribed into cDNA using the PrimeScript™ RT Reagent Kit (TaKaRa, RR037A). Quantitative PCR was performed on a real‐time fluorescence detection system using TB Green^®^ Premix Ex Taq™ II (TaKaRa, RR820A) with cDNA as the template. Each reaction was run in triplicate, and β-actin was used as the internal control. Relative gene expression levels were calculated by the 2^^−ΔΔCt^ method. Primer sequences are listed in **Supplementary Table 1**.

### 2.3 Western blot

Cells were lysed in RIPA buffer on ice for 5 minutes and then sonicated to shear genomic DNA. Tissue samples were homogenized in RIPA buffer using a tissue grinder, incubated on ice for 1 hour, and centrifuged to collect the supernatant. Protein concentrations were determined with the BCA Protein Assay Kit (Solarbio, PC0020). Equal amounts of protein were separated by SDS–PAGE and transferred onto membranes at a constant current of 200 mA for 1.5 hours. Membranes were blocked with 5% nonfat milk for 1 hour at room temperature, incubated with primary antibodies overnight at 4 °C, then with HRP-conjugated secondary antibodies for 1 hour at room temperature. Antibody binding was detected using an enhanced chemiluminescence (ECL) kit (Proteintech, PK10002). Bands were visualized on a chemiluminescence imaging system and quantified with ImageJ software. Antibodies used in this study:-RSPO2 (Cusabio, CSB-PA020551GA01HU; 1:2000)-PTBP1 (Proteintech, 12582-1-AP; 1:5000)-FLAG (Cell Signaling Technology, 14793; 1:1000)- TRIM21 (Proteintech, 12108-1-AP; 1:5000) - HA (Cell Signaling Technology, 3724; 1:1000)- FSP1 (Proteintech, 20886-1-AP; 1:1000)-GAPDH (Proteintech, 60004-1-Ig; 1:50,000) Secondary antibodies: - HRP-conjugated goat anti-mouse IgG (H+L) (Proteintech, SA00001-1; 1:10,000)- HRP-conjugated goat anti-rabbit IgG (H+L) (Proteintech, SA00001-2; 1:10,000).

### 2.4 Immunohistochemical staining

Tissue sections were deparaffinized in xylene and rehydrated through a graded ethanol series. Antigen retrieval was performed by heating the sections in citrate buffer (pH 6.0) for 15 minutes in a microwave oven. Endogenous peroxidase activity was blocked with 3% hydrogen peroxide for 10 minutes at room temperature. Sections were then incubated with 5% bovine serum albumin for 30 minutes at room temperature to block non-specific binding, followed by overnight incubation with primary antibody against RSPO2 at 4 °C. After washing with PBS, sections were incubated with HRP-conjugated secondary antibody for 1 hour at room temperature. Antibody binding was visualized using DAB substrate, and sections were counterstained with hematoxylin. Finally, sections were dehydrated, cleared, and mounted with neutral resin. All images were captured using a light microscope.

### 2.5 Cell transfection

For overexpression, the coding sequences (CDS) of RSPO2, PTBP1 and FSP1 were each cloned into pcDNA3.1-Flag, pcDNA3.1-HA and pcDNA3.1-Myc vectors (denoted OE-RSPO2, OE-PTBP1 and OE-FSP1). Cells were seeded in 6-well plates and, at ~70–80% confluence, transfected with 2 µg plasmid DNA using Lipofectamine 3000 Reagent (Invitrogen, L3000008). Briefly, plasmid DNA and P3000 reagent were diluted in serum-free medium, mixed with Lipofectamine 3000, incubated at room temperature for 20 min, and then added dropwise to the cells. After 48 h, cells were harvested for further assays. For knockdown, shRNA oligonucleotides targeting RSPO2 and TRIM21 were synthesized (Beijing Qingke Biology Co., Ltd.) and cloned into the pLKO.1 vector. Lentiviral particles were produced by co-transfecting HEK293T cells with sh-RSPO2 or sh-TRIM21 plasmid, pSPAX2 (Addgene, #12260) and pMD2.G (Addgene, #12259) using Lipofectamine 3000. At 24–48 h post-transfection, supernatants were collected, filtered through a 0.45 µm filter, and concentrated by ultracentrifugation. MCF-7 and MDA-MB-231 cells were then infected with the lentivirus in the presence of 10 µg/mL polybrene (Santa Cruz Biotechnology, sc-134220) for 48h. Medium was replaced with fresh complete medium before downstream experiments. Primer and shRNA sequences are listed in **Supplementary Table 2**.

### 2.6 EdU proliferation assay

MCF-7 and MDA-MB-231 cells were seeded into 24-well plates pre-loaded with glass coverslips (WHB, T3421219). After cells had adhered, plasmid transfection was performed as described above. Forty-eight hours post-transfection, cells on coverslips were fixed and then subjected to 5-ethynyl-2′-deoxyuridine (EdU) incorporation analysis using the BeyoClick™ EdU-555 Cell Proliferation Kit (Beyotime, C0075S) strictly according to the manufacturer’s protocol. Finally, fluorescent images were acquired with an inverted fluorescence microscope (Olympus, Tokyo, Japan).

### 2.7 Cell cycle analysis

MCF-7 and MDA-MB-231 cells were harvested and fixed in 75% ethanol at –20 °C for at least 2 hours. Following fixation, cells were washed with PBS and stained with propidium iodide (PI; 10 μg/mL; Yeasen, 40710ES03) for 30 minutes at room temperature in the dark. DNA content was then analyzed on a FACSCalibur flow cytometer (BD Biosciences, San Jose, CA, USA), and cell cycle phase distribution was determined using FlowJo software.

### 2.8 Transwell invasion assay

Cell invasive capacity was measured using 24-well Transwell inserts with an 8 µm pore-size polycarbonate membrane (Corning, 3422). Inserts were coated on the upper surface with 100 µl of 1 mg/mL Matrigel (Corning, 354234) diluted in cold serum-free medium and incubated at 37 °C for 1h. Transfected cells were trypsinized, resuspended in serum-free medium, and 200 µL of a 1.5×10^^6^ cells/mL suspension was added to the upper chamber. The lower chamber was filled with medium containing 10% FBS as a chemoattractant. After 24 h incubation at 37 °C in a 5% CO_2_ incubator, non-invading cells on the upper membrane surface were removed with a cotton swab. Invaded cells on the underside were fixed in 4% paraformaldehyde for 30 min, stained with 1% crystal violet (Beyotime, C0121) for 5 min, rinsed with distilled water, and air-dried. Representative fields were photographed under an inverted microscope (Olympus, Tokyo, Japan), and invaded cells were quantified using ImageJ software.

### 2.9 Wound healing assay

Transfected MCF-7 and MDA-MB-231 cells were seeded into 6-well plates. Once the monolayer reached 100% confluence, a straight scratch was made through the cell layer using a sterile 100 µL pipette tip to create a wound. Culture medium was then replaced with medium containing 2% FBS, and cells were incubated at 37 °C in a 5% CO_2_ atmosphere. Representative images of cell migration into the wound area were captured at 0 h and 24 h using an inverted optical microscope.

### 2.10 Immunoprecipitation

Whole-cell lysates were prepared in NP-40 lysis buffer (Beyotime, P0013F) supplemented with 50 µg/mL phenylmethylsulfonyl fluoride (PMSF) and a protease inhibitor cocktail. Lysates were cleared by centrifugation at 12,000×g for 15 min at 4 °C, and the supernatants were incubated with antibody-conjugated Protein A/G magnetic beads overnight at 4 °C. After five washes with IP wash buffer, bound proteins were eluted by boiling in SDS-PAGE loading buffer and analyzed by Western blot.

### 2.11 Silver staining and mass spectrometry identification

Flag-RSPO2 was transiently expressed in MCF-7 cells, and Flag-tagged protein complexes were immunoprecipitated using an anti-Flag antibody to enrich for RSPO2-interacting partners. The immunoprecipitated proteins were resolved by SDS-PAGE, and gels were processed with the Beyotime Fast Silver Stain Kit (P0017S) according to the manufacturer’s protocol. Briefly, gels were fixed, sensitized, silver-stained, and developed; once protein bands became distinct, the staining reaction was terminated with the provided stop solution. Gels were then rinsed in ultrapure water and imaged on a gel documentation system. Differentially stained bands were excised and subjected to liquid chromatography–tandem mass spectrometry (LC-MS/MS) for protein identification.

### 2.12 Immunofluorescence

MCF-7 and MDA-MB-231 cells were washed with PBS, fixed in 4% paraformaldehyde for 5 min, and then washed three times with PBS. Cells were blocked in blocking buffer at room temperature for 2 h and subsequently incubated overnight at 4 °C with primary antibodies. After two additional PBS washes, cells were incubated with the appropriate fluorescently labeled secondary antibodies for 1h at room temperature and counterstained with DAPI (Beyotime, C1002). Fluorescent images were acquired on a Zeiss LSM800 confocal microscope (Oberkochen, Germany). The primary antibodies used were: RSPO2 (CUSABIO, CSB-PA020551GA01HU; 1:200). PTBP1 (Proteintech, 12582-1-AP; 1:200).TRIM21 (Proteintech, 12108-1-AP; 1:200).

### 2.13 Cycloheximide chase assay

MCF-7 and MDA-MB-231 cells transfected with either an RSPO2 overexpression plasmid or a control empty vector were seeded in six-well plates. When the cultures reached approximately 80% confluence, cells were synchronized and treated with cycloheximide (CHX; MCE, HY-12320) at a final concentration of 30 μM. Cell samples were harvested at 0, 2, 4, and 8 h after CHX addition, and PTBP1 protein levels were assessed by Western blot analysis.

### 2.14 RNA immunoprecipitation

RIP was carried out using the EZ-Magna RIP^®^ RNA-Binding Protein Immunoprecipitation Kit (Millipore, 17-701) according to the manufacturer’s protocol. Briefly, cells were washed with PBS and then lysed in RIP lysis buffer supplemented with protease inhibitors and RNase inhibitors. Genomic DNA was removed by DNase treatment, and an aliquot of the cleared lysate was taken as the Input control. The remaining lysate was incubated overnight at 4 °C with rotation in the presence of either an anti-PTBP1 antibody or control IgG. The following day, protein A/G magnetic beads were added and samples were incubated at 4 °C for 1 h to capture RNA–protein complexes. After digestion with proteinase K (Takara, 9034), RNA bound to the immunoprecipitated complexes was extracted with TRIzol reagent. Purified RNA was reverse-transcribed, and enrichment of the target transcripts was quantified by RT-qPCR. Relative enrichment was calculated using the Input sample as reference.

### 2.15 Luciferase reporter assay

The 3′UTR of FSP1 was cloned into the psiCHECK-2 vector. Cells were co-transfected with this construct using Lipofectamine 3000 Reagent (Invitrogen, L3000008). Forty-eight hours after transfection, luciferase activities were measured using the Dual-Luciferase Reporter Assay Kit (Vazyme, DL101-01). Firefly luciferase signals were normalized to those of Renilla luciferase.

### 2.16 Actinomycin D chase assay

MCF-7 and MDA-MB-231 cells were seeded in 6-well plates and, following transfection, cultured to ~80% confluence. Cells were then treated with actinomycin D (MCE, HY-17559) for 0, 3, 6, or 9 h. At each time point, cells were harvested and total RNA was extracted using TRIzol reagent. cDNA was synthesized by reverse transcription, and FSP1 mRNA levels were quantified by qPCR. Relative expression at each time point was calculated using the 2^^–ΔΔCt^ method, with the 0 h sample serving as reference.

### 2.17 ROS staining

Reactive oxygen species (ROS) levels were assessed using the ROS Detection Kit (Beyotime, S0033M). Following the designated treatments, cells were incubated with 10 μM DCFH-DA in fresh culture medium at 37 °C for 30 min in the dark. Cells were then washed three times with PBS and immediately imaged under a fluorescence microscope (excitation/emission wavelengths: 488/525 nm).

### 2.18 MDA assay

Malondialdehyde (MDA) levels were measured using the MDA Assay Kit (Beyotime, S0131M). Briefly, cells were harvested and washed twice with cold PBS, then lysed in the kit-provided lysis buffer on ice. Lysates were centrifuged at 10 000 g for 10 min at 4 °C. The resulting supernatant was mixed 1:1 with the MDA reaction working solution and heated at 100 °C for 15 min. After cooling to room temperature, samples were centrifuged at 1000g for 10 min, and the final supernatant was transferred to a 96 well plate. Absorbance was read at 532 nm using a microplate reader (Synergy HT, BioTek, Broadview, IL).

### 2.19 GSH assay

Reduced glutathione (GSH) levels were measured using the GSH Content Assay Kit (Solarbio, BC1175). After transfection, MCF-7 and MDA-MB-231 cells were harvested and mixed with the kit’s protein precipitant to extract proteins. The protein suspensions were subjected to two freeze–thaw cycles alternating between liquid nitrogen and a 37 °C water bath. Samples were then centrifuged at 8 000 g for 10 min, and 20 µl of the resulting supernatant was transferred to a 96‐well plate. The GSH assay working solution was added, and the plate was incubated at 25 °C for 2 min. Absorbance was measured at 412 nm using a microplate reader.

### 2.20 Fe^2+^ assay

The intracellular ferrous ion (Fe2+) content was determined using the Ferrous Ion Assay Kit (Solarbio, BC5415). Briefly, 1×10^^7^ cells were harvested and resuspended in 0.5 mL of the kit’s extraction reagent, then lysed by sonication on ice. Lysates were centrifuged at 10 000 g for 10 min at 4 °C, and the supernatant was kept on ice for analysis. Fe2+ levels were measured by recording the absorbance at 593 nm using a microplate reader (Synergy HT, BioTek, Broadview, IL), and concentrations were calculated based on a ferrous ion standard curve.

### 2.21 Animal experiments

All procedures involving animals were approved by the Institutional Animal Care and Use Committee of our institution. Ten female BALB/c nude mice (6 weeks old) were purchased from Hunan Silaike Jingda Experimental Animal Co., Ltd. and housed under SPF conditions (22–25 °C, 50–60% humidity, 12 h light/12 h dark cycle) with ad libitum access to food and water. After a 1-week acclimation period, mice were randomized into two groups (n=5 per group). The experimental group received MCF-7 cells stably overexpressing RSPO2 (via lentiviral transduction), while the control group received MCF-7 cells transduced with empty vector. Beginning on Day 1, each mouse was injected subcutaneously in the right axillary region with 1×10^7 cells in 100 µL PBS. Tumor dimensions—long diameter (L) and short diameter (W)—were measured every three days starting on Day 7 using calipers, and tumor volume was calculated as: V (mm³) = (L × W²)/2. Tumor growth curves were plotted from these measurements. On Day 28, mice were humanely euthanized by cervical dislocation under deep anesthesia induced by isoflurane, and tumors were excised, photographed, and weighed.

### 2.22 Statistical analysis

All experiments were independently repeated at least three times, and data are presented as mean ± standard deviation (SD). All statistical analyses were performed using GraphPad Prism 9.0 software. Prior to parametric tests, the normality of data distribution was assessed using the Shapiro-Wilk test, and homogeneity of variances was evaluated using Levene’s test. For data that met the assumptions of normal distribution and homogeneity of variance, comparisons between two groups were performed using two-tailed Student’s t-test, and comparisons among multiple groups were analyzed using one-way or two-way analysis of variance (ANOVA) followed by Tukey’s *post hoc* test for multiple comparisons. For data that did not meet the assumptions of normality or equal variance, non-parametric tests were applied (Mann-Whitney U test for two-group comparisons, and Kruskal-Wallis test for multiple-group comparisons).Statistical analyses for densitometric quantification of Western blots, cell proliferation, migration, invasion, tumor volume, and other data in this study were performed following the methods described above. The specific statistical tests used, the number of biological replicates, and the statistical significance (*p* values) for each experiment are indicated in the corresponding figure legends. A *p* value of less than 0.05 was considered statistically significant (*p < 0.05, **p < 0.01, ***p < 0.001).

## 3 Results

### 3.1 RSPO2 is downregulated in breast cancer and suppresses its malignant phenotype

**Figure 1:**
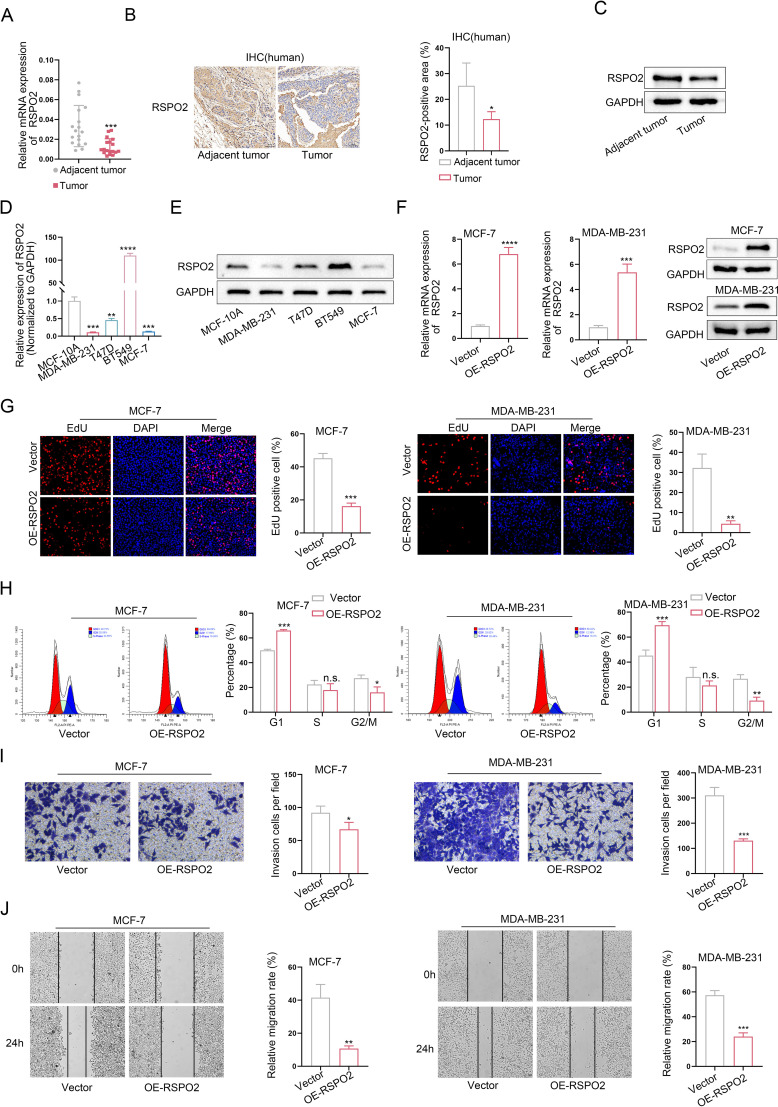
RSPO2 is downregulated in breast cancer and inhibits its malignant phenotypes. **(A)** RT-qPCR analysis of RSPO2 mRNA expression levels in breast cancer tissues and adjacent normal tissues. **(B)** Immunohistochemistry (IHC) and **(C)** Western blot analysis of RSPO2 protein expression levels in breast cancer tissues and adjacent normal tissues. **(D)** RT-qPCR analysis of RSPO2 mRNA expression levels in breast cancer cell lines and normal breast epithelial cells. **(E)** Western blot analysis of RSPO2 protein expression levels in breast cancer cell lines and normal breast epithelial cells. **(F)** Validation of RSPO2 expression efficiency by RT-qPCR and Western blot following its overexpression. **(G)** EdU assays showing the effect of RSPO2 overexpression or knockdown on the proliferation of breast cancer cells. **(H)** Flow cytometry analysis of cell cycle distribution in breast cancer cells following RSPO2 overexpression or knockdown. **(I)** Transwell assays assessing the invasion of breast cancer cells following RSPO2 overexpression or knockdown. **(J)** Wound-healing assays assessing the migration of breast cancer cells following RSPO2 overexpression or knockdown. *p<0.05; **p<0.01; ***p<0.001; ****p<0.0001; n.s. : not significant.

To investigate the role of RSPO2 in the pathogenesis and progression of breast cancer, we first examined its expression levels in clinical breast cancer samples and cell lines. RT-qPCR analysis of tissue samples revealed that RSPO2 mRNA expression was significantly lower in breast cancer tissues compared to adjacent normal tissues (**Figure 1A**). Furthermore, both immunohistochemistry (IHC) and Western blot analyses confirmed that RSPO2 protein expression was markedly downregulated in cancerous tissues (**Figures 1B, C**). Further validation in a panel of breast cancer cell lines showed that RSPO2 was expressed at low levels in MCF-7 and MDA-MB-231 cells, at a high level in BT549 cells, whereas its expression was significantly increased in T47D cells compared to the control (**Figures 1D, E**). These findings suggest that RSPO2 may play distinct roles in different subtypes of breast cancer. Based on these findings, we further investigated whether RSPO2 exerts a tumor-suppressive function in the RSPO2-low cell lines, MCF-7 and MDA-MB-231. First, we cloned the RSPO2 coding sequence into the pcDNA3.1-Flag vector to construct a Flag-RSPO2 overexpression plasmid. Concurrently, we designed and synthesized shRNAs targeting RSPO2. Validation by RT-qPCR and Western blot confirmed the high efficiency of both overexpression and knockdown (**Figure 1F**; **Supplementary Figure 1A**). We then transfected the respective constructs into the breast cancer cells for subsequent functional assays. Results from EdU and flow cytometry assays showed that overexpression of RSPO2 significantly inhibited cell proliferation and induced G1-phase cell cycle arrest (**Figures 1G, H**), whereas knockdown of RSPO2 had the opposite effects (**Supplementary Figures 1B, C**). Concurrently, Transwell and wound-healing assays demonstrated that RSPO2 overexpression suppressed the invasion and migration capabilities of breast cancer cells (**Figures 1I, J**), while its knockdown enhanced these malignant phenotypes (**Supplementary Figures 1D, E**). Taken together, these results suggest that RSPO2 functions as a tumor suppressor in breast cancer and that its overexpression can inhibit the malignant phenotypes of tumor cells.

### 3.2 RSPO2 inhibits the malignant progression of breast cancer cells by promoting the degradation of the PTBP1 protein

**Figure 2:**
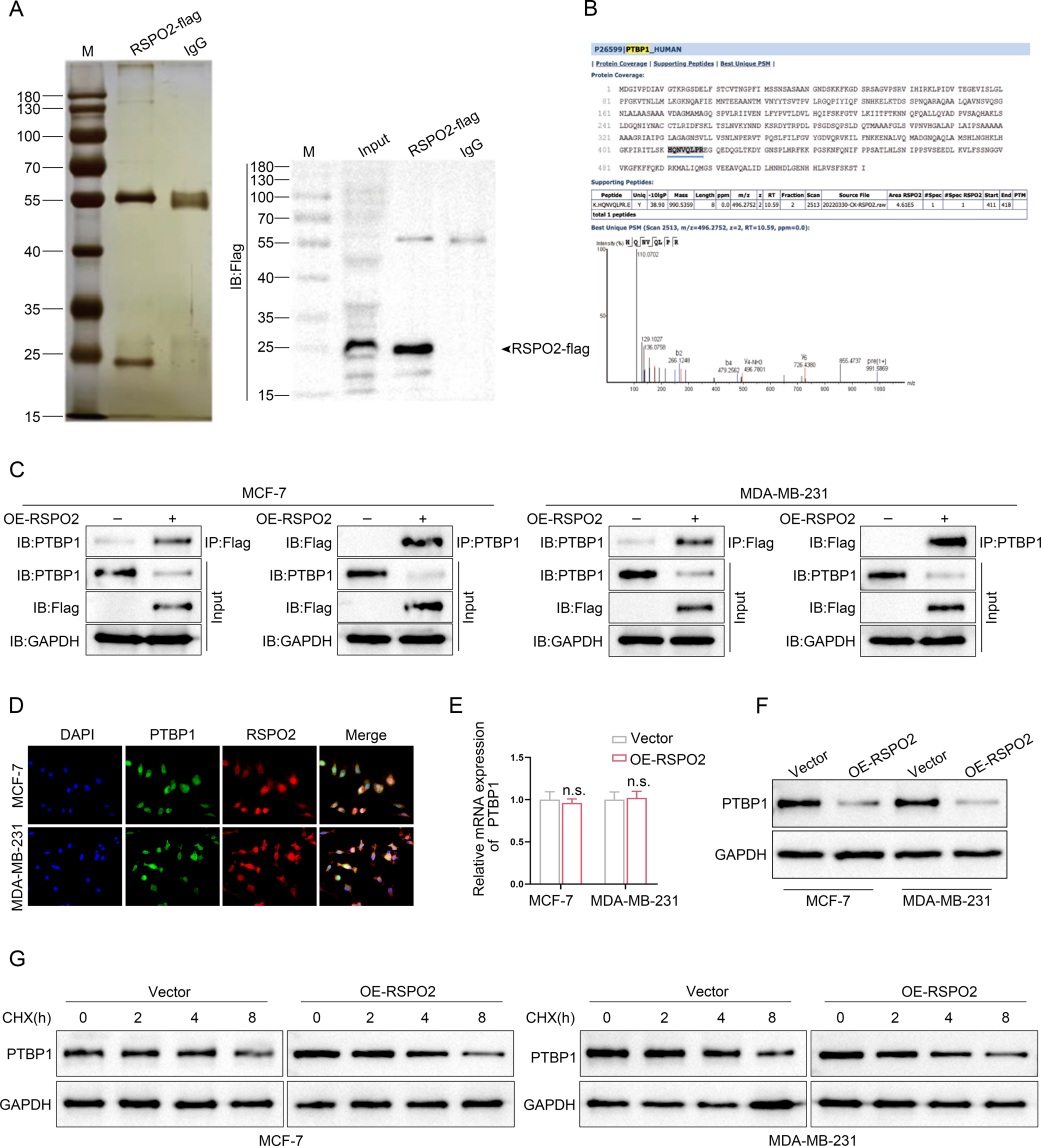
RSPO2 inhibits the malignant progression of breast cancer cells by promoting PTBP1 protein degradation. **(A)** Co-immunoprecipitation (Co-IP) was performed in RSPO2-overexpressing MCF-7 cells to pull down RSPO2-interacting proteins, followed by silver staining of the protein gel. **(B)** Mass spectrometry (MS) analysis identifying proteins that interact with RSPO2 (representative mass spectrum shown). **(C)** Reciprocal Co-IP assays validating the interaction between RSPO2 and PTBP1. **(D)** Immunofluorescence (IF) staining validating the colocalization of RSPO2 and PTBP1. **(E)** qRT-PCR analysis of PTBP1 mRNA expression in cells overexpressing RSPO2. **(F)** Western blot analysis of PTBP1 protein expression in cells overexpressing RSPO2. **(G)** Cycloheximide (CHX) chase assay to determine the protein stability of PTBP1 in cells overexpressing RSPO2. n.s. : not significant.

To elucidate the mechanism underlying the role of RSPO2 in breast cancer progression, we performed a co-immunoprecipitation (Co-IP) assay in MCF-7 cells transfected with Flag-RSPO2. The immunoprecipitated complexes were visualized by silver staining (**Figure 2A**), and subsequent mass spectrometry analysis revealed multiple potential interacting proteins. Among these candidates, PTBP1 was identified and chosen for follow-up studies (**Figure 2B**). PTBP1, an RNA-binding protein (RBP), is known to be overexpressed in multiple cancers, such as breast cancer, glioma, and colorectal cancer, where it facilitates tumorigenesis by modulating processes like energy metabolism, proliferation, apoptosis, and metastasis (8). Of note, PTBP1 has been reported to sustain the growth and malignancy of breast cancer cells through the activation of the PTEN/Akt pathway and the regulation of autophagy (11). Therefore, to confirm the interaction between RSPO2 and PTBP1, we conducted reciprocal co-immunoprecipitation (Co-IP) assays. A specific interaction between RSPO2 and PTBP1 was demonstrated (**Figure 2C**) and further validated by immunofluorescence (IF) experiments (**Figure 2D**). We next sought to determine how RSPO2 regulates PTBP1. Following the overexpression of RSPO2 in breast cancer cells, RT-qPCR analysis revealed that PTBP1 mRNA levels were unaffected (**Figure 2E**). In contrast, Western blot analysis showed a significant reduction in PTBP1 protein levels (**Figure 2F**), suggesting a post-transcriptional regulatory mechanism. Collectively, these findings indicated that RSPO2 regulates PTBP1 post-transcriptionally. To elucidate the underlying mechanism, we performed a cycloheximide (CHX) chase assay to assess PTBP1 protein stability. We found that the overexpression of RSPO2 significantly enhanced the degradation rate of PTBP1, demonstrating that RSPO2 downregulates PTBP1 by promoting its protein instability (**Figure 2G**). To determine whether the effects of RSPO2 on breast cancer cell function are mediated by PTBP1, we conducted a series of rescue experiments. We first constructed a PTBP1 overexpression plasmid and confirmed its successful expression by RT-qPCR and Western blot analysis (**Supplementary Figure 2A**). Next, we co-transfected breast cancer cells with both the RSPO2 and PTBP1 overexpression plasmids. The results of the EdU assay revealed that the anti-proliferative effect of RSPO2 was significantly rescued by the overexpression of PTBP1 (**Supplementary Figure 2B**). Similarly, Transwell and wound-healing assays showed that the co-expression of PTBP1 also counteracted the suppressive effects of RSPO2 on cell invasion and migration (**Supplementary Figures 2C, D**). Taken together, these findings demonstrate that RSPO2 suppresses breast cancer malignancy by directly binding to PTBP1 and promoting its subsequent degradation.

### 3.3 RSPO2 recruits TRIM21 to mediate the ubiquitin-dependent degradation of PTBP1

**Figure 3:**
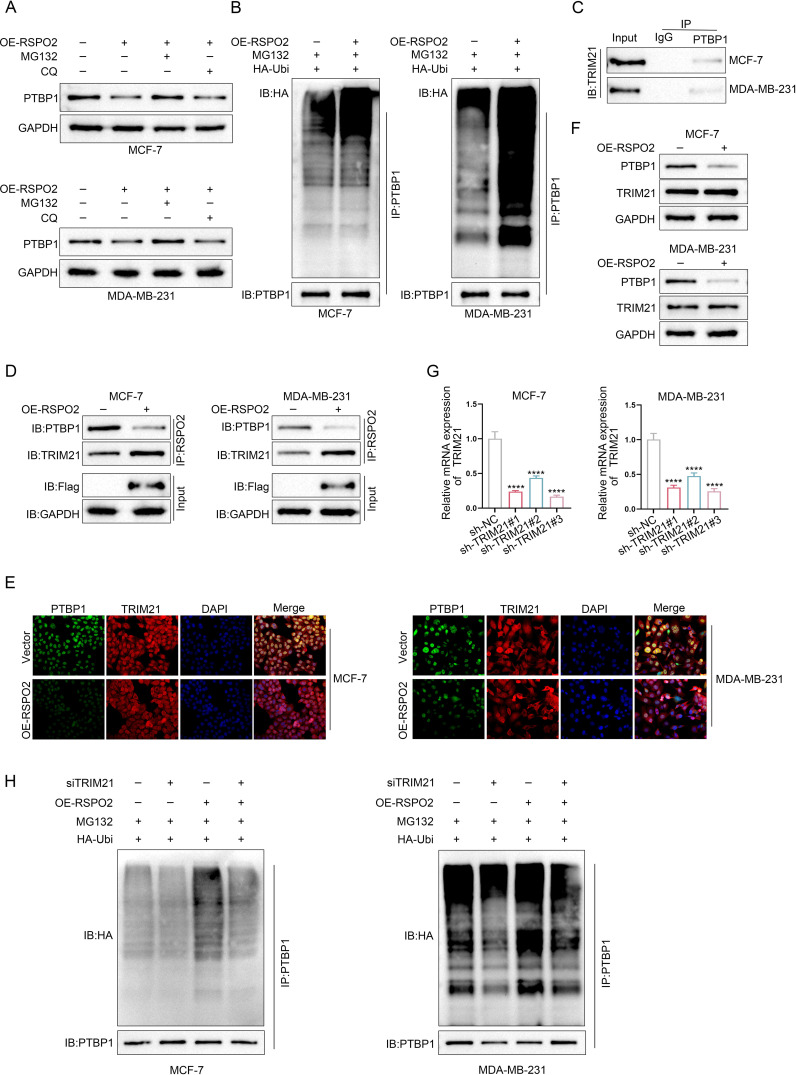
RSPO2 promotes the ubiquitination and degradation of PTBP1 by recruiting TRIM21. **(A)** Western blot analysis of PTBP1 expression in RSPO2-overexpressing breast cancer cells treated with the proteasome inhibitor MG132 or the autophagy inhibitor chloroquine (CQ). **(B)** Co-IP and Western blot analysis of PTBP1 ubiquitination levels in breast cancer cells co-transfected with RSPO2 and HA-tagged ubiquitin (HA-Ubi). **(C)** Co-IP assay validating the interaction between TRIM21 and PTBP1 in breast cancer cells. **(D)** Co-IP assay showing that RSPO2 interacts with both PTBP1 and TRIM21. **(E)** IF staining showing the colocalization of PTBP1 and TRIM21 upon RSPO2 overexpression. **(F)** Western blot analysis of TRIM21 and PTBP1 protein levels following RSPO2 overexpression. **(G)** RT-qPCR analysis validating the knockdown efficiency of siTRIM21 in breast cancer cells. **(H)** Co-IP and Western blot analysis of PTBP1 ubiquitination levels in RSPO2-overexpressing cells with or without TRIM21 knockdown, treated with MG132. **** p < 0.0001.

To elucidate the specific mechanism governing the degradation of PTBP1 by RSPO2, we treated RSPO2-overexpressing breast cancer cells with the proteasome inhibitor MG132 and the autophagy inhibitor chloroquine (CQ), respectively. Western blot analysis revealed that the downregulation of PTBP1 caused by RSPO2 overexpression was abolished by MG132 treatment. In contrast, CQ treatment did not alter PTBP1 protein levels (**Figure 3A**). These findings suggest that RSPO2 promotes PTBP1 degradation predominantly in a proteasome-dependent manner. Based on these observations, we postulated that RSPO2 facilitates PTBP1 degradation by promoting its ubiquitination. To test this, we performed an *in vivo* ubiquitination assay by co-transfecting breast cancer cells with plasmids for RSPO2 and HA-ubiquitin (HA-Ub) in the presence of MG132. Following immunoprecipitation of PTBP1, Western blotting revealed a marked increase in its ubiquitination levels upon RSPO2 overexpression (**Figure 3B**). These results provide further evidence that RSPO2 directs PTBP1 for degradation through the ubiquitin-proteasome system. Given that the E3 ubiquitin ligase TRIM21 was previously reported to bind the RRM 2 and RRM 3 domains of PTBP1 and mediate its degradation (18), we postulated that RSPO2 acts by recruiting TRIM21 to PTBP1. To test this hypothesis, we first validated the endogenous interaction between TRIM21 and PTBP1 in breast cancer cells by reciprocal co-immunoprecipitation (Co-IP) (**Figure 3C**). Next, to probe the role of RSPO2, we performed Co-IP assays in cells overexpressing RSPO2. The results demonstrated that RSPO2 interacts with both PTBP1 and TRIM21 (**Figure 3D**), indicating the formation of a ternary complex among these three proteins. Immunofluorescence analysis revealed a significant reduction in PTBP1 and TRIM21 colocalization following RSPO2 overexpression (**Figure 3E**). Consistent with our previous findings, Western blotting confirmed that RSPO2 overexpression decreased PTBP1 levels without affecting the expression of TRIM21 itself (**Figure 3F**). To directly test the functional requirement of TRIM21 in this pathway, we knocked down its expression using a validated shRNA (**Figure 3G**). We then performed an *in vivo* ubiquitination assay by overexpressing RSPO2 in these TRIM21-depleted cells to measure the effect on PTBP1 ubiquitination via Co-IP. Results from the ubiquitination assay revealed that knockdown of TRIM21 markedly reduced PTBP1 ubiquitination. Critically, this effect could not be reversed by overexpressing RSPO2, demonstrating that TRIM21 is required for the RSPO2-mediated degradation of PTBP1 (**Figure 3H**). Collectively, these findings support a model in which RSPO2 facilitates PTBP1 degradation by recruiting the E3 ligase TRIM21 to its substrate.

### 3.4 PTBP1 inhibits ferroptosis by binding to the FSP1 3’ UTR, which enhances its mRNA stability

**Figure 4:**
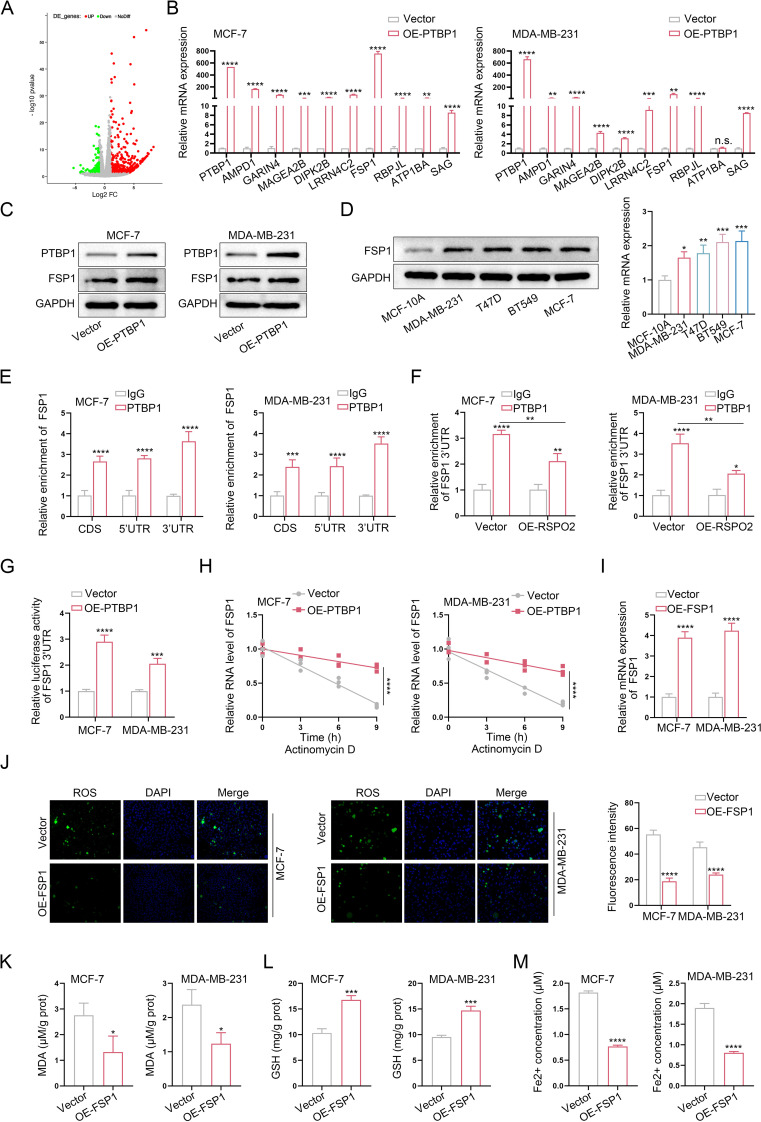
PTBP1 inhibits ferroptosis by binding to the 3’UTR of FSP1 mRNA to enhance its stability. **(A)** Volcano plot of differentially expressed genes from RNA sequencing (RNA-seq) of control cells versus PTBP1-overexpressing cells. **(B)** RT-qPCR validation of the mRNA expression levels of the top 10 candidate genes. **(C)** Western blot validation of the protein expression levels of the top 10 candidate genes. **(D)** RT-qPCR and Western blot results showing high expression of FSP1 in breast cancer cells. **(E)** RNA immunoprecipitation (RIP)-qPCR analysis validating the binding between PTBP1 and FSP1 mRNA. **(F)** Dual-luciferase reporter assay validating the binding of PTBP1 to the 3’UTR of FSP1. **(G)** Actinomycin D chase assay to determine the mRNA stability of FSP1 in PTBP1-overexpressing breast cancer cells. **(H)** RT-qPCR analysis validating the overexpression efficiency of FSP1. **(I–L)** Measurement of cellular ROS **(I)**, MDA **(J)**, GSH **(K)**, and Fe2+ **(L)** levels in breast cancer cells overexpressing FSP1. *p<0.05; **p<0.01; ***p<0.001; ****p<0.0001; n.s. : not significant.

To elucidate the downstream network regulated by the RSPO2/PTBP1 axis, we conducted RNA-sequencing (RNA-seq) analysis comparing cells overexpressing PTBP1 with control cells (**Figure 4A**). From the differentially expressed genes identified, we selected the top 10 most significantly upregulated candidates for further investigation. Subsequent validation by RT-qPCR confirmed our sequencing data and highlighted FSP1 as the most prominently upregulated gene (**Figure 4B**). Furthermore, Western blotting confirmed that overexpressing PTBP1 led to a significant increase in FSP1 protein levels (**Figure 4C**), prompting us to select FSP1 for subsequent study. PRG3, also known as FSP1, is a well-established ferroptosis suppressor first identified as a p53-responsive gene and known to play a critical role in multiple cancers, such as breast cancer. Building on this, we next compared FSP1 expression between normal breast epithelial cells and breast cancer cell lines. Both RT-qPCR and Western blot analyses showed that FSP1 expression was significantly elevated in breast cancer cells relative to normal cells (**Figure 4D**), suggesting a potential oncogenic role for FSP1 in the progression of breast cancer. As an RNA-binding protein, PTBP1 is a central player in tumor biology, regulating key post-transcriptional events including alternative splicing, mRNA stability, and translation (19). To determine whether PTBP1 directly binds FSP1 mRNA, we performed a RIP-qPCR assay using primers targeting the FSP1 5’ UTR, CDS, and 3’ UTR. The results showed a significant enrichment of PTBP1 on the FSP1 3’ UTR. Notably, this interaction was significantly diminished upon RSPO2 overexpression (**Figure 4E**). This finding is consistent with previous reports showing that PTBP1 can stabilize target mRNAs by binding to specific 3’ UTR elements like AU-rich elements (AREs), a mechanism known to promote the expression of oncogenic proteins and drive malignant phenotypes such as tumor angiogenesis, migration, invasion, and drug resistance (20). These findings led us to hypothesize that PTBP1 directly binds the FSP1 mRNA 3’ UTR to enhance its stability and upregulate its expression. To test this, we first cloned the FSP1 3’ UTR into a psiCHECK2 dual-luciferase reporter vector. Co-expression of this reporter with a PTBP1 plasmid in breast cancer cells resulted in a significant increase in luciferase activity (**Figure 4F**), confirming a specific interaction. Second, we performed an Actinomycin D chase assay, which revealed that PTBP1 overexpression significantly extended the half-life of FSP1 mRNA (**Figure 4G**), thus validating its stabilizing effect. Collectively, these data demonstrate that PTBP1 enhances the stability of FSP1 mRNA through direct binding to its 3’ UTR. Since FSP1 is a well-established ferroptosis suppressor, we next assessed its functional role in breast cancer. After confirming the efficacy of an FSP1 overexpression plasmid (**Figure 4H**), we found that FSP1 overexpression decreased cellular ROS levels (**Figure 4I**). Consistent with ferroptosis inhibition, this also resulted in a significant reduction in MDA and Fe2+ levels and an elevation in GSH (**Figures 4J–L**). In summary, our results establish a clear axis where PTBP1 inhibits ferroptosis in breast cancer cells by stabilizing its downstream target, FSP1 mRNA.

### 3.5 The RSPO2/PTBP1/FSP1 axis attenuates the malignant progression of breast cancer via the promotion of ferroptosis

**Figure 5:**
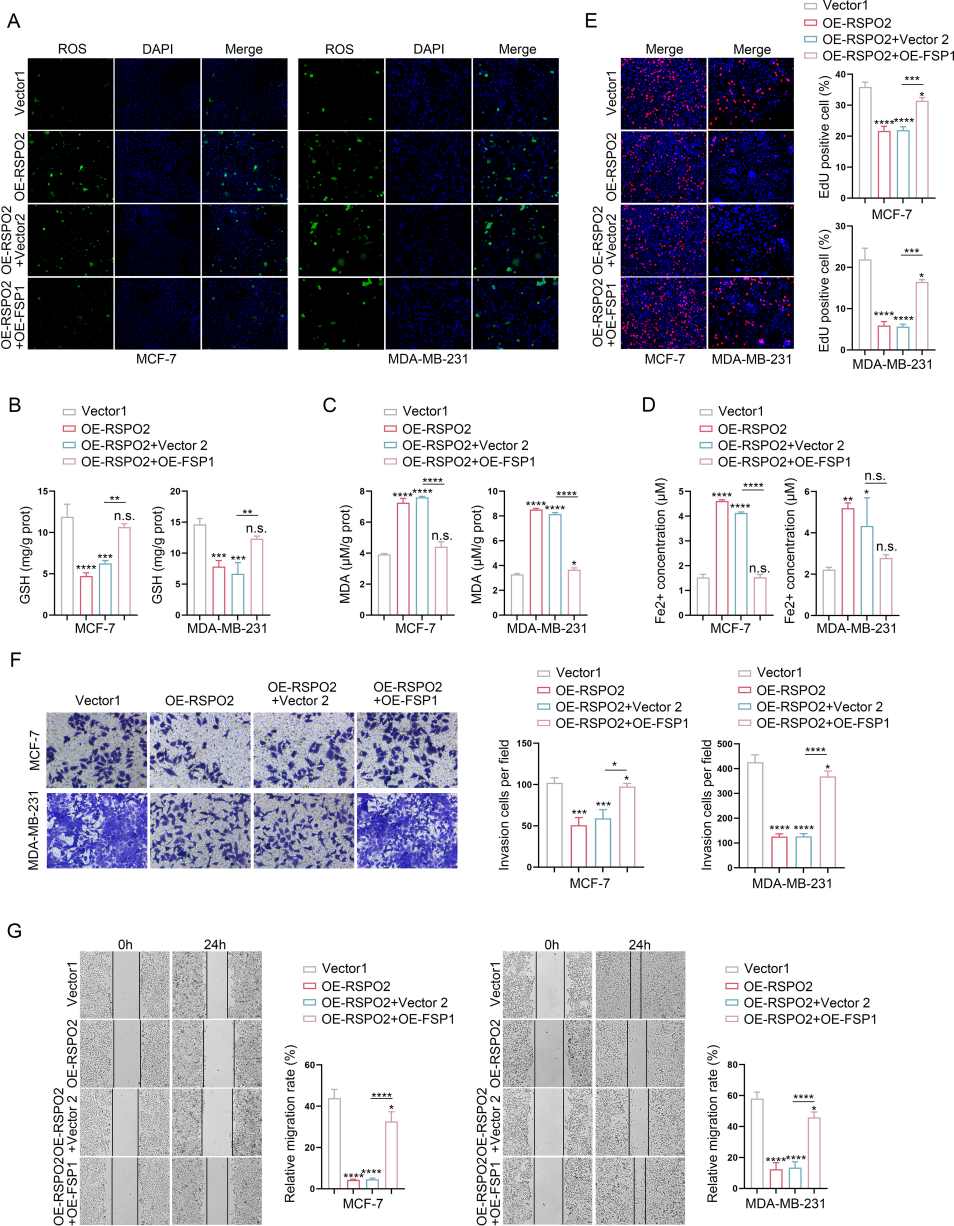
The RSPO2/PTBP1/FSP1 axis delays the malignant progression of breast cancer by promoting ferroptosis. **(A)** IF analysis of cellular ROS levels in breast cancer cells co-overexpressing RSPO2 and FSP1. **(B–D)** Measurement of cellular GSH **(B)**, MDA **(C)**, and Fe2+ **(D)** levels in breast cancer cells co-overexpressing RSPO2 and FSP1. **(E–G)** Analysis of cell proliferation [EdU, **(E)**], migration [wound-healing assay, **(F)**], and invasion [Transwell assay, **(G)**] in breast cancer cells co-overexpressing RSPO2 and FSP1. *p<0.05; **p<0.01; ***p<0.001; ****p<0.0001; n.s. : not significant.

To further confirm the regulatory function of the RSPO2/PTBP1/FSP1 axis in ferroptosis, we conducted a rescue experiment. In breast cancer cells overexpressing RSPO2, which exhibited elevated ROS levels, subsequent overexpression of FSP1 successfully reversed this phenotype (**Figure 5A**). Similarly, the decrease in GSH and the increase in MDA and Fe2+ levels caused by RSPO2 overexpression were all significantly rescued by the reintroduction of FSP1 (**Figures 5B–D**). These findings establish that RSPO2 promotes ferroptosis through the PTBP1/FSP1 axis. We next explored the impact of this pathway on breast cancer malignancy. Functionally, RSPO2 overexpression significantly suppressed the proliferation (EdU assay), migration (Wound-healing assays, and invasion (Transwell and wound-healing assays) of breast cancer cells. Crucially, these suppressive effects were all effectively reversed by the co-expression of FSP1 (**Figures 5E–G**). To further validate that RSPO2 exerts its tumor-suppressive effects through inducing ferroptosis, we employed the specific ferroptosis inhibitor Ferrostatin-1 (Fer-1) in RSPO2-overexpressing breast cancer cells. Treatment with Fer-1 effectively reversed the inhibitory effects of RSPO2 overexpression on cell proliferation, migration, and invasion (**Supplementary Figures 3A–C**). Taken together, these data reveal a clear mechanism where RSPO2 inhibits breast cancer progression by suppressing the PTBP1/FSP1 axis, thereby triggering ferroptosis.

### 3.6 The RSPO2/PTBP1/FSP1 axis inhibits breast cancer growth *in vivo*

**Figure 6:**
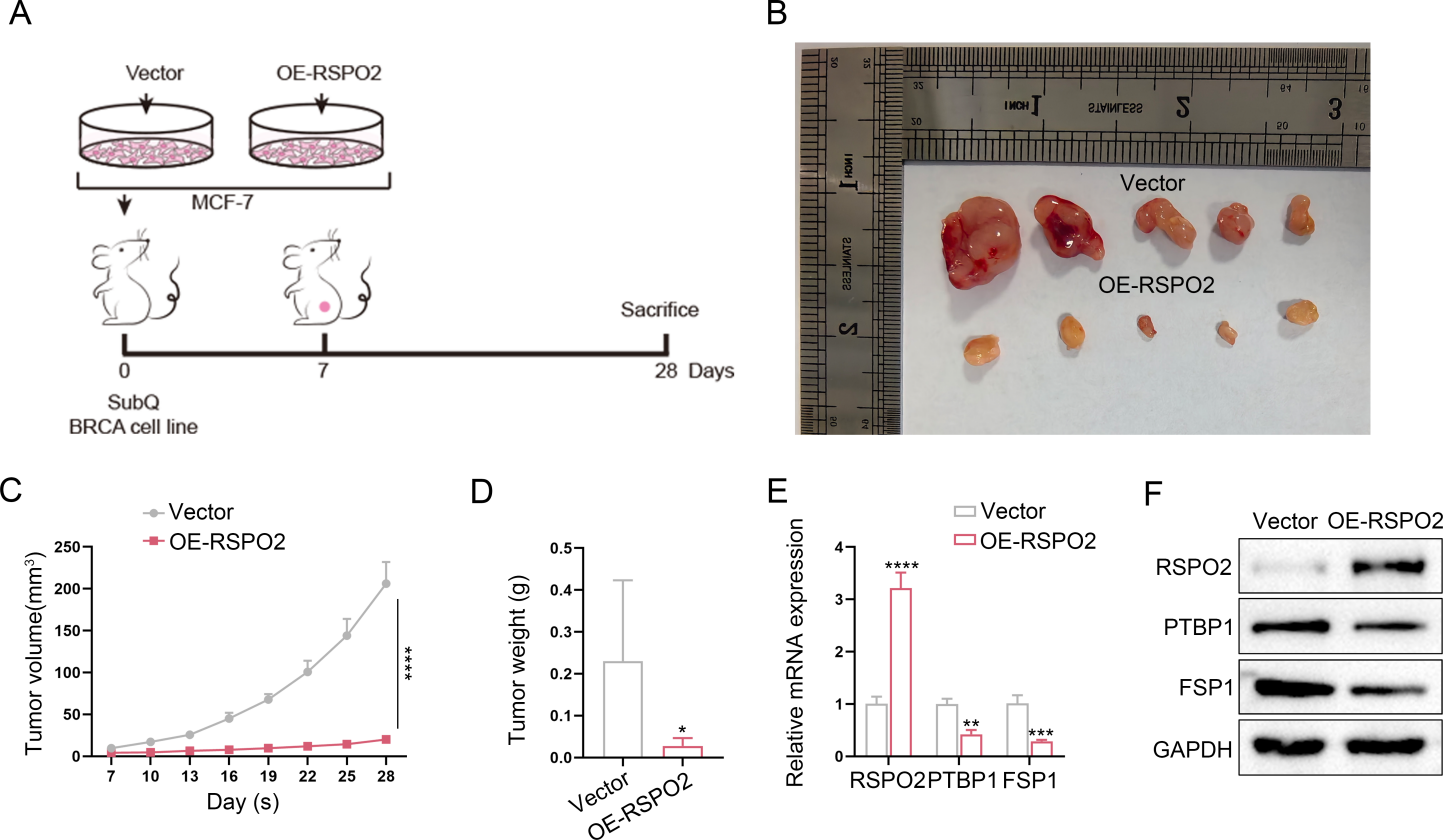
*In vivo* experiments confirm that the RSPO2/PTBP1/FSP1 axis suppresses breast cancer growth. **(A)** Schematic diagram of the mouse xenograft model. Experimental groups: Vector group, OE-RSPO2 group. **(B)** Representative images of excised tumors and measurements of tumor dimensions. **(C)** Tumor volume growth curves. **(D)** Final tumor weights. **(E)** RT-qPCR analysis of RSPO2, PTBP1, and FSP1 expression in tumor tissues. **(F)** Western blot analysis of RSPO2, PTBP1, and FSP1 expression in tumor tissues. (Representative tumor specimens are shown. **Figure 1B** is from the first batch of animal experiments, which exhibited larger inter-individual variation in tumor volume; **Figure 1C** is from a second batch of animals with more homogeneous baseline characteristics. All statistical analyses were performed on the complete tumor volume datasets). * p < 0.05, ** p < 0.01, *** p < 0.001, **** p < 0.0001.

To assess the *in vivo* function of the RSPO2/PTBP1/FSP1 axis, we generated xenograft tumors in nude mice using MCF-7 cells stably overexpressing either RSPO2 or an empty vector control (**Figure 6A**). Notably, RSPO2 overexpression significantly suppressed tumor growth, as evidenced by smaller tumor volumes and lower weights compared to controls (**Figures 6B–D**). Mechanistically, tumors from the RSPO2-overexpressing group displayed downregulated expression of both PTBP1 and FSP1 at the mRNA and protein levels (**Figures 6E, F**). Taken together, these *in vivo* data demonstrate that RSPO2 impedes breast cancer progression by downregulating the PTBP1/FSP1 axis, thereby solidifying its crucial role in breast cancer oncogenesis and progression.

## 4 Discussion

Breast cancer, characterized by high heterogeneity and complex metabolic reprogramming, remains a leading cause of cancer-related mortality worldwide. Although the classical Wnt signaling enhancer R-spondin 2 (RSPO2) has been extensively studied in various developmental and pathological contexts, its specific role and downstream mechanisms in the metabolic regulation of breast cancer remain largely unclear. In this study, we identified RSPO2 as a potential tumor suppressor in breast cancer and elucidated a novel RSPO2/PTBP1/FSP1 axis. We demonstrated that RSPO2 promotes the degradation of PTBP1 by recruiting the E3 ubiquitin ligase TRIM21. This, in turn, destabilizes the mRNA of the ferroptosis suppressor FSP1, ultimately inducing ferroptosis in breast cancer cells and inhibiting their malignant progression.

Emerging evidence indicates that RSPO2 has a pronounced functional dichotomy, with its expression and role varying significantly across different cancers. Indeed, a comprehensive analysis of multiple tumor types revealed that RSPO2 is downregulated in 11 malignancies, such as bladder and colon adenocarcinoma, yet upregulated in a minority of cancers, while its prognostic value is highly tumor-specific (5). This context-dependent duality is likely linked to tissue-specific Wnt pathway regulation and variations in the tumor microenvironment (21). These findings underscore that RSPO2 cannot be simply classified as an “oncogene” or a “tumor suppressor”; rather, its biological function is dictated by its specific cellular and molecular context. Our own findings, which show low RSPO2 expression in MCF-7 and MDA-MB-231 cells but high expression in BT549 cells, exemplify this molecular heterogeneity inherent to breast cancer. It is noteworthy that some literature reports a contradictory role for RSPO2, showing its specific upregulation in triple-negative breast cancer (TNBC)—especially in the highly aggressive metaplastic subtype—where it correlates with poor patient prognosis (22). This implies that the high RSPO2 level in BT549 cells could reflect its potential oncogenic function within such aggressive contexts. Therefore, to elucidate the function of RSPO2 in a broader range of breast cancers, we centered our investigation on MCF-7 and MDA-MB-231 cells, which exhibit low endogenous RSPO2 expression. Our functional assays demonstrated that ectopic expression of RSPO2 markedly suppressed the proliferation, migration, and invasion of both cell lines. These findings were further validated *in vivo*, where RSPO2 overexpression significantly inhibited breast cancer growth.

It should be noted that although RSPO2 is classically considered a secreted ligand that activates Wnt/β-catenin signaling by binding to cell membrane receptors, in this study we detected RSPO2 protein in cell lysates, and immunofluorescence showed its co-localization with PTBP1 and TRIM21 in the cytoplasm. Therefore, we propose that under overexpression conditions, a portion of RSPO2 protein is retained intracellularly and acts as a scaffold protein to facilitate TRIM21-mediated ubiquitination and degradation of PTBP1. This intracellular function of RSPO2 is independent of its classical secreted pathway and may represent a novel tumor-suppressive mechanism beyond its canonical regulation of Wnt signaling.

As a multifunctional RNA-binding protein (RBP), PTBP1 is well-established as an oncogenic driver in numerous cancers, including breast cancer, where its overexpression modulates pre-mRNA splicing, mRNA stability, and translation to promote malignancy (19). For instance, PTBP1 facilitates breast cancer cell proliferation and survival by suppressing PTEN expression, thereby activating the Akt pathway and inducing protective autophagy (11). Moreover, a distinct mechanism in triple-negative breast cancer involves the downregulation of miR-152, which derepresses SLC7A5 and leads to E2F1-mediated transcriptional activation of PTBP1. This promotes the critical PKM1-to-PKM2 metabolic switch, coordinately enhancing amino acid metabolism and glycolysis to fuel tumor growth and confer therapeutic resistance (23). Crucially, the stability of the PTBP1 protein is also subject to tight regulation. A key example is the deubiquitinase USP46, which stabilizes PTBP1 to sustain a glycolytic phenotype. Consequently, USP46 knockdown triggers the K48-linked ubiquitination and proteasomal degradation of PTBP1, leading to a decreased PKM2/PKM1 ratio, suppressed glycolytic flux, and enhanced sensitization to the anti-tumor effects of tamoxifen (11). However, the precise mechanisms governing PTBP1 function and its upstream regulation in breast cancer remain largely unexplored. Here, we identify RSPO2 as a novel and critical upstream regulator of PTBP1. We first established a specific interaction between RSPO2 and PTBP1 using Co-IP, mass spectrometry, and immunofluorescence assays. Crucially, we discovered that RSPO2 overexpression accelerated PTBP1 protein turnover via the ubiquitin-proteasome system without affecting its mRNA levels. This finding prompted us to consider the E3 ubiquitin ligase TRIM21, which was previously shown to bind the RRM2 and RRM3 domains of PTBP1 to mediate its degradation (18). We therefore hypothesized that RSPO2 acts as a scaffold to recruit TRIM21 to PTBP1. Indeed, our experiments confirmed that RSPO2, PTBP1, and TRIM21 can form a ternary complex. To functionally validate this mechanism, we found that RSPO2 overexpression failed to rescue the loss of PTBP1 ubiquitination in TRIM21-depleted cells, conclusively demonstrating that RSPO2 mediates PTBP1 degradation in a TRIM21-dependent manner.

Ferroptosis, a form of regulated cell death triggered by iron-dependent lipid peroxidation, has recently emerged as a promising strategy for anti-cancer therapy (24). In the present study, our transcriptome sequencing analysis, confirmed by experimental validation, revealed FSP1 as a key downstream target of PTBP1. FSP1 is the central mediator of the non-canonical ferroptosis defense pathway. This system operates independently of the canonical GPX4/GSH axis, conferring resistance to ferroptosis by reducing coenzyme Q10 to neutralize toxic lipid peroxides (25). As a pivotal RNA-binding protein, PTBP1 steers tumor progression by modulating the stability of its target mRNAs. For instance, PTBP1 has been shown to bind the 3’UTR of PGK1 mRNA, enhancing its stability and upregulating its expression to drive gastric cancer cell migration (26). In another context, the circular RNA hsa_circ_0005358 acts as a sponge for PTBP1, preventing its stabilizing effect on CDCP1 mRNA and consequently suppressing CDCP1 translation and cervical cancer metastasis (27). Conversely, PTBP1 can also destabilize its targets; it binds to KLF9 mRNA to reduce its stability, thereby fostering colorectal cancer (CRC) cell stemness and chemoresistance to cisplatin (DDP) (28). These findings collectively pointed to a model where PTBP1 could regulate FSP1 via a similar post-transcriptional mechanism. Indeed, we discovered that PTBP1 directly binds to the 3’UTR of FSP1 mRNA, thereby enhancing its stability and increasing FSP1 expression. Functionally, this upregulation of FSP1 suppresses ferroptosis by reducing intracellular levels of ROS, MDA, and Fe2+. Our findings are corroborated by literature reporting that PTBP1 can also suppress ferroptosis in endometrial cancer cells by binding the 5’UTR of SLC7A11 mRNA to enhance its stability and maintain protein expression (29). To functionally validate our proposed axis, we demonstrated that the pro-ferroptotic effect of RSPO2 overexpression was significantly abrogated upon co-expression of FSP1. In summary, this study delineates a complete signaling pathway: RSPO2 triggers the ubiquitin-mediated degradation of PTBP1, leading to the destabilization of FSP1 mRNA. This reduction in FSP1 levels sensitizes cells to ferroptosis, thereby suppressing the malignant progression of breast cancer. In addition, our transcriptomic sequencing also identified multiple other differentially expressed genes regulated by the RSPO2-PTBP1 axis. These genes may similarly play important functions in breast cancer and warrant systematic validation in future studies.

Nevertheless, several limitations of this study should be acknowledged. First, our cohort of breast cancer cell lines and clinical samples did not fully encompass the diversity of major molecular subtypes. Moreover, our *in vivo* studies were primarily based on subcutaneous xenograft models, which poorly recapitulate the native microenvironment of orthotopic breast tumors. These factors constrained a more thorough elucidation of the subtype-specific roles of RSPO2 and its precise pathophysiological significance. Second, while we identified TRIM21 as a key E3 ubiquitin ligase for PTBP1 degradation, the specific structural domains and residues mediating the RSPO2/PTBP1/TRIM21 ternary complex formation remain to be elucidated. It also remains an open question whether other E3 ligases are involved in regulating PTBP1 stability. Finally, PTBP1 governs an intricate post-transcriptional network. Our focus on the FSP1-mediated ferroptosis pathway does not preclude the possibility that other downstream targets may act in concert to exert a broader tumor-suppressive effect. Future studies will be designed to address these limitations by expanding our validation pipeline with more sophisticated animal models, diverse cellular systems, and a broader range of clinical specimens. Such efforts will not only deepen our mechanistic understanding of the “RSPO2/PTBP1/FSP1” tumor-suppressive axis but will also lay a stronger theoretical groundwork for the development of novel breast cancer therapies targeting this pathway.

## 5 Conclusions

In conclusion, our study unveils a novel tumor-suppressive role for RSPO2 in breast cancer and delineates the underlying molecular mechanism. We demonstrate that RSPO2 orchestrates the ubiquitin-mediated degradation of PTBP1 by recruiting the E3 ligase TRIM21. This downregulation of PTBP1, in turn, destabilizes the mRNA of its downstream target FSP1, leading to reduced FSP1 expression. The resulting cellular state is sensitized to ferroptosis, ultimately inhibiting the malignant progression of breast cancer. This discovery of the RSPO2/PTBP1/FSP1 axis provides a critical link between RSPO2 and ferroptosis and establishes a strong rationale for developing novel therapeutic strategies that target this pathway in breast cancer.

## Institutional review board statement

All procedures performed in studies involving human participants were approved by the Research Ethics Committee of Qinghai University Affiliated Hospital ((approval no. P-SL-2024-029; approval date: 25 July 2024) and in accordance with the 1964 Declaration of Helsinki and its later amendments or comparable ethical standards. Written informed consent was obtained for each participant. All animal experiments were performed with the approval of the Research Ethics Committee of Qinghai University Affiliated Hospital (approval no. P-SL-2024-029; approval date: 25 July 2024) and the procedures for Care and Use of Laboratory Animals in cancer research.

## Data Availability

The data presented in this study are deposited in the NCBI Sequence Read Archive (SRA) repository, under BioProject accession number PRJNA1470379.

## Supplementary material

Supplementary Figure 1 **(A)** Validation of RSPO2 knockdown efficiency by RT-qPCR and Western blot in breast cancer cells. **(B)** EdU assays showing the effect of RSPO2 knockdown on the proliferation of breast cancer cells. **(C)** Flow cytometry analysis of cell cycle distribution in breast cancer cells following RSPO2 knockdown. **(D)** Transwell assays assessing the invasion of breast cancer cells following RSPO2 knockdown. **(E)** Wound-healing assays assessing the migration of breast cancer cells following RSPO2 knockdown. **p*<0.05; ***p*<0.01; ****p*<0.001; *****p*<0.0001; n.s.: not significant.

Supplementary Figure 2 **(A)** Validation of PTBP1 expression efficiency by RT-qPCR and Western blot. **(B–D)** Rescue experiments were performed by co-overexpressing PTBP1 in RSPO2-overexpressing cells. Cell proliferation [EdU, **(B)**], invasion [Transwell, **(C)**], and migration [wound-healing assay, **(D)**] were assessed. **p*<0.05; ***p*<0.01; ****p*<0.001; *****p*<0.0001.

Supplementary Figure 3 **(A–C)** Analysis of cell proliferation [EdU, **(A)**], invasion [Transwell assay, **(B)**], and migration [wound-healing assay, **(C)**] in RSPO2-overexpressing breast cancer cells treated with Ferrostatin-1 (Fer-1). ***p*<0.01; ****p*<0.001; *****p*<0.0001; n.s.: not significant.
