# Supplementary figures and images for: RSPO2-induced ferroptosis via PTBP1-mediated FSP1 mRNA decay suppresses breast cancer progression
Source: Front Oncol. 2026 Jun 9;16:1813451. doi: 10.3389/fonc.2026.1813451 (PMC13286778; doi:10.3389/fonc.2026.1813451)

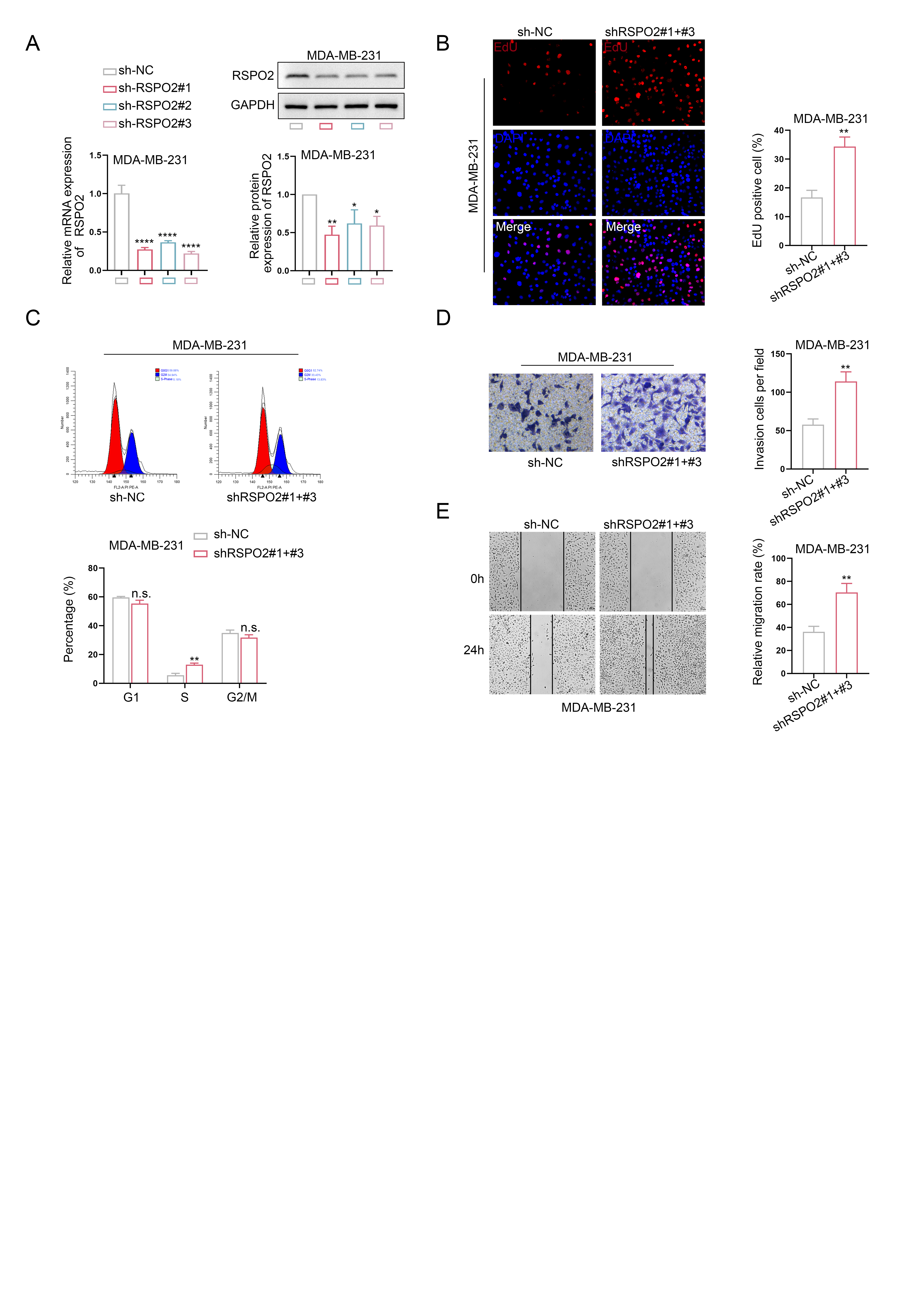

Supplement: Supplementary Figure 1 — (A) Validation of RSPO2 knockdown efficiency by RT-qPCR and Western blot in breast cancer cells. (B) EdU assays showing the effect of RSPO2 knockdown on the proliferation of breast cancer cells. (C) Flow cytometry analysis of cell cycle distribution in breast cancer cells following RSPO2 knockdown. (D) Transwell assays assessing the invasion of breast cancer cells following RSPO2 knockdown. (E) Wound-healing assays assessing the migration of breast cancer cells following RSPO2 knockdown. *p<0.05; **p<0.01; ***p<0.001; ****p<0.0001; n.s.: not significant. [file Image1.tif]

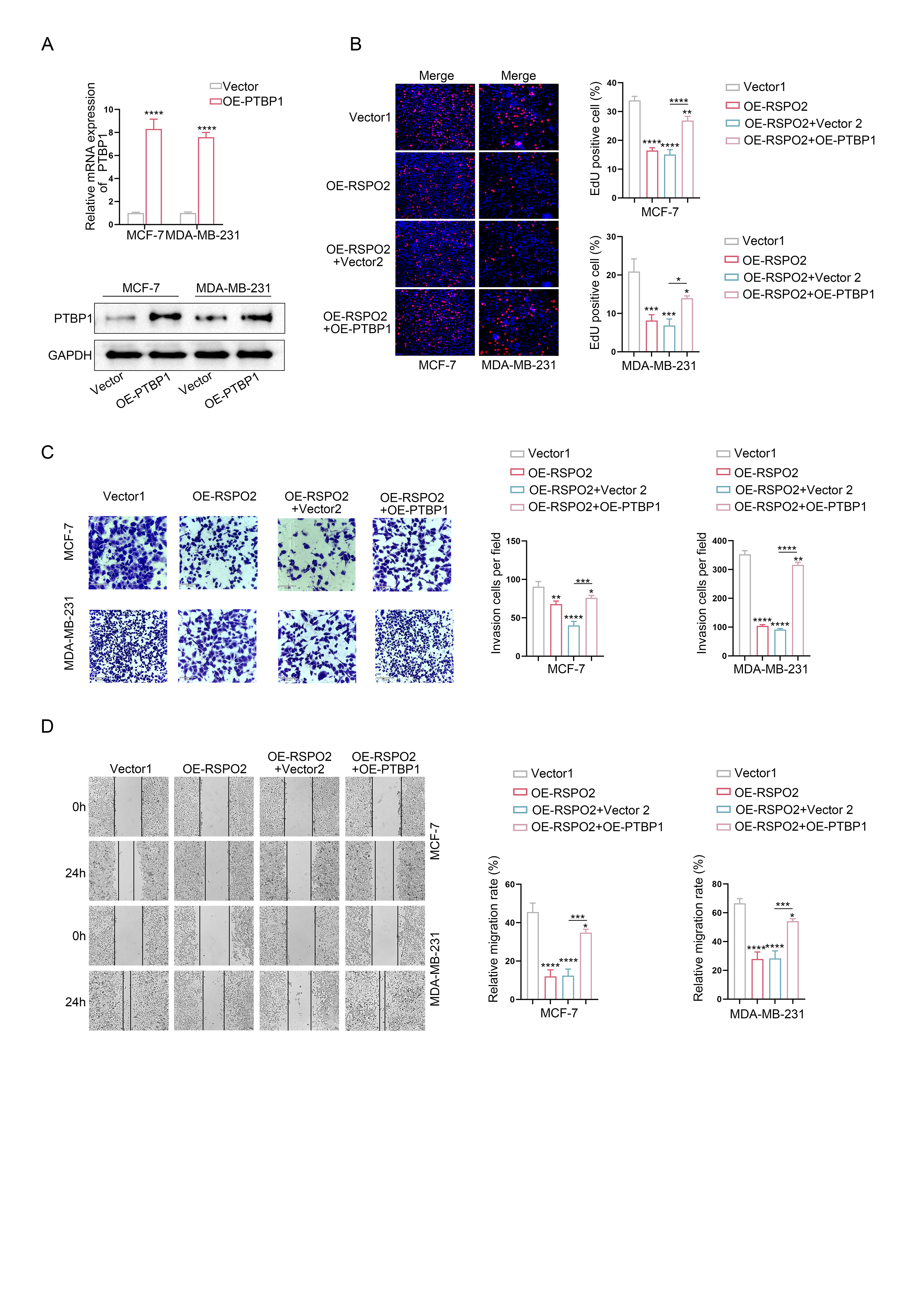

Supplement: Supplementary Figure 2 — (A) Validation of PTBP1 expression efficiency by RT-qPCR and Western blot. (B–D) Rescue experiments were performed by co-overexpressing PTBP1 in RSPO2-overexpressing cells. Cell proliferation [EdU, (B)], invasion [Transwell, (C)], and migration [wound-healing assay, (D)] were assessed. *p<0.05; **p<0.01; ***p<0.001; ****p<0.0001. [file Image2.tif]

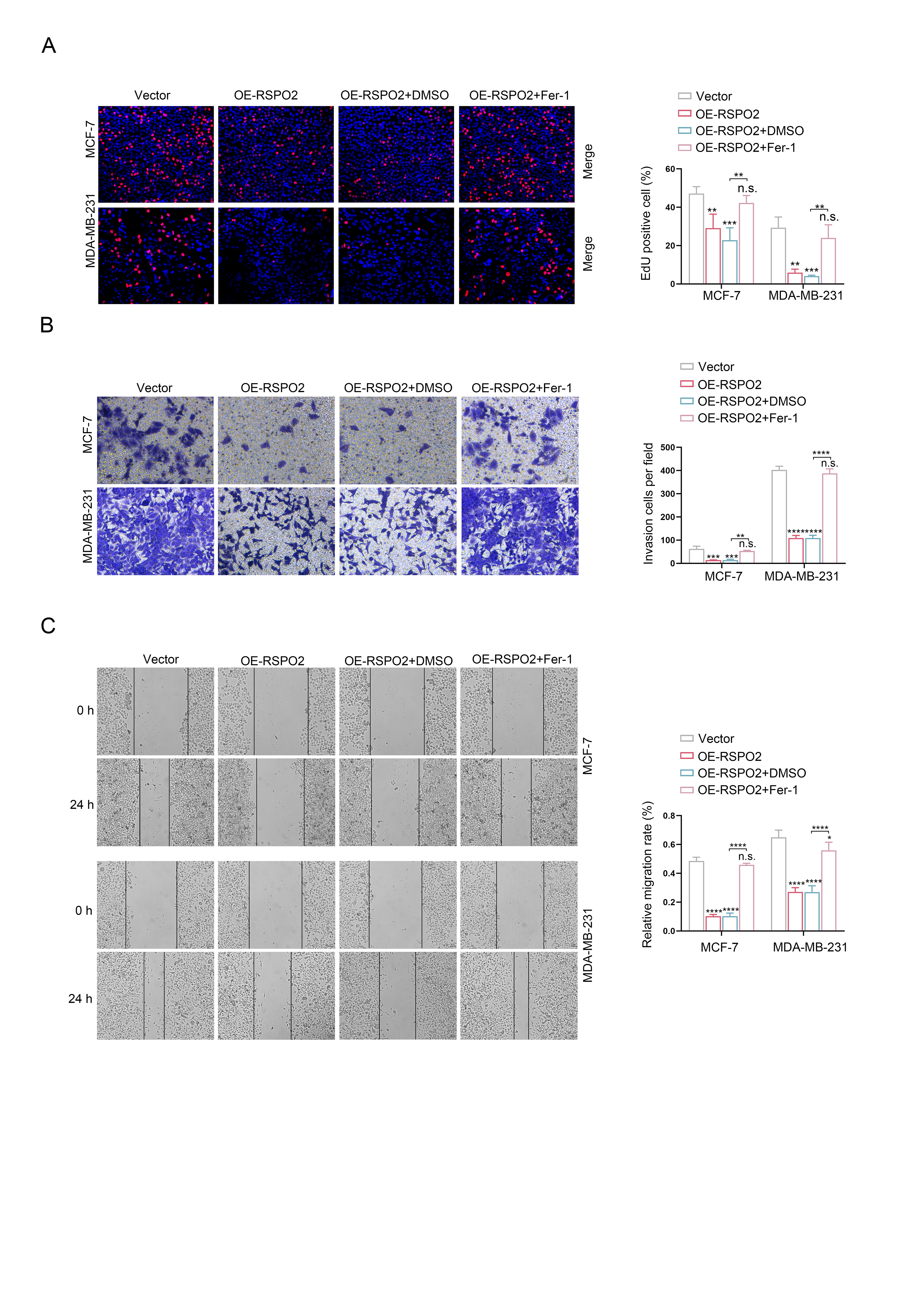

Supplement: Supplementary Figure 3 — (A–C) Analysis of cell proliferation [EdU, (A)], invasion [Transwell assay, (B)], and migration [wound-healing assay, (C)] in RSPO2-overexpressing breast cancer cells treated with Ferrostatin-1 (Fer-1). **p<0.01; ***p<0.001; ****p<0.0001; n.s.: not significant. [file Image3.tif]
