# Supplementary material for: RSPO2-induced ferroptosis via PTBP1-mediated FSP1 mRNA decay suppresses breast cancer progression
Source: Front Oncol. 2026 Jun 9;16:1813451. doi: 10.3389/fonc.2026.1813451 (PMC13286778; doi:10.3389/fonc.2026.1813451)
Supplement: Supplementary file 4 [file Table1.docx]

Table 1.RT-qPCR Primer Sequences

| Gene | Sequence | |
| --- | --- | --- |
| hsa_RSPO2 | Forward (5’-3’)  Reverse (5’-3’) | AGAGGCCGTTGCTTTGATGA  TTGCCGTGTTCTGGTTTCCA |
| hsa_PTBP1 | Forward (5’-3’)  Reverse (5’-3’) | AGAACGCCCTAGTGCAGATG  GTTGCCGTAGTCCTTGGTCA |
| hsa_TRIM21 | Forward (5’-3’)  Reverse (5’-3’) | GCAGGAGTTGGCTGAGAAGT  GCTGCTCCCTCTCATCCTTC |
| hsa_AMPD1 | Forward (5’-3’)  Reverse (5’-3’) | GGCCACATGTTCTCCTCCAA  CATGAGATGGGTGAGGGCTC |
| hsa_GARIN4 | Forward (5’-3’)  Reverse (5’-3’) | CGCAAGGCAGCAAAGAACTT  TCATCCCGTGTGTCAAGAGC |
| hsa_MAGEA2B | Forward (5’-3’)  Reverse (5’-3’) | TCCGAGTTCCAAGCAGCAAT  ACCTCGATGCCAAAGACCAG |
| hsa_DIPK2B | Forward (5’-3’)  Reverse (5’-3’) | TCACCAGGGCCAGAGAGAAC  GGAGCTGCATGTGAAGGTTTG |
| hsa_LRRN4C2 | Forward (5’-3’)  Reverse (5’-3’) | TTCTCCCCGGTCCTCCACTA  CTTCAGCCCCTTCAGTTCGG |
| hsa_FSP1 | Forward (5’-3’)  Reverse (5’-3’) | CCCTTCATGCTGGTGGACAT  ATCCCCACTACTAGCCCCTG |
| hsa_RBPJL | Forward (5’-3’)  Reverse (5’-3’) | ACAGGGCTCTGCTTAACGAC  GTCCCCAAACCACACCTTGA |
| hsa_ATP1B4 | Forward (5’-3’)  Reverse (5’-3’) | GAAGAGGGTCAAGGTCAGCC  TGATCACAGCAGCCAAGGAG |
| hsa_SAG | Forward (5’-3’)  Reverse (5’-3’) | GAAGCTCACAGTGTCAGGCT  CTCTCTTCCCCTCCTCAGCT |
| hsa_β-actin | Forward (5’-3’)  Reverse (5’-3’) | CCACCATGTACCCTGGCATT  CGGACTCGTCATACTCCTGC |
| mmu_RSPO2 | Forward (5’-3’)  Reverse (5’-3’) | AGCGAATGGGGAACGTGTAG  CTCCTGGACAGTGCCTCATG |
| mmu_PTBP1 | Forward (5’-3’)  Reverse (5’-3’) | AGTGCGCATTACACTGTCCA  GGTTTCTTGAAGCGGTGCAG |
| mmu_FSP1 | Forward (5’-3’)  Reverse (5’-3’) | ATGTCCCCTTCATGCTGGTG  AGATGTGAGAAGGGCAAGGC |
| Mmu_β-actin | Forward (5’-3’)  Reverse (5’-3’) | CAACACCCCAGCCATGTAC  CACGATTTCCCTCTCAGCT |
