# Supplementary material for: RSPO2-induced ferroptosis via PTBP1-mediated FSP1 mRNA decay suppresses breast cancer progression
Source: Front Oncol. 2026 Jun 9;16:1813451. doi: 10.3389/fonc.2026.1813451 (PMC13286778; doi:10.3389/fonc.2026.1813451)
Supplement: Supplementary file 5 [file Table2.docx]

Table 2.RT-qPCR Primer Sequences

| Gene | Forward primer（5’-3’） | Reverse primer（5’-3’） |
| --- | --- | --- |
| pcDNA-3.1-Flag-RSPO2 | GCTAGCATGCAGTTTCGCCTTTTCTCC | GGTACCTTATTGGTTAGCTCTGTCTGTAGCTAGG |
| pcDNA-3.1-HA-PTBP1 | GCTAGCATGGACGGCATTGTCCCAG | GGTACCCTAGATGGTGGACTTGGAGAAGGA |
| pcDNA-3.1-Myc-FSP1 | GGTACCATGGGGTCCCAGGTCTCGG | GGATCCTCAAGGTGGAGACTGCCTCATG |
| sh-RSPO2#1 | CCGGCGAGCTAGTTATGTATCAAATCTCGAGATTTGATACATAACTAGCTCGTTTTTG | AATTCAAAAACGAGCTAGTTATGTATCAAATCTCGAGATTTGATACATAACTAGCTCG |
| sh-RSPO2#2 | CCGGGCAAGGGTTGTTTGTCTTGTTCTCGAGAACAAGACAAACAACCCTTGCTTTTTG | AATTCAAAAAGCAAGGGTTGTTTGTCTTGTTCTCGAGAACAAGACAAACAACCCTTGC |
| sh-RSPO2#3 | CCGGCCATTGCTGAATCCAGGAGATCTCGAGATCTCCTGGATTCAGCAATGGTTTTTG | AATTCAAAAACCATTGCTGAATCCAGGAGATCTCGAGATCTCCTGGATTCAGCAATGG |
| sh-TRIM21#1 | CCGGTGAGAAGTTGGAAGTGGAAATCTCGAGATTTCCACTTCCAACTTCTCATTTTTG | AATTCAAAAATGAGAAGTTGGAAGTGGAAATCTCGAGATTTCCACTTCCAACTTCTCA |
| sh-TRIM21#2 | CCGGTGGCATGGTCTCCTTCTACAACTCGAGTTGTAGAAGGAGACCATGCCATTTTTG | TGGCATGGTCTCCTTCTACAACTCGAGTTGTAGAAGGAGACCATGCCA |
| sh-TRIM21#3 | CCGGGAGTTGGCTGAGAAGTTGGAACTCGAGTTCCAACTTCTCAGCCAACTCTTTTTG | AATTCAAAAAGAGTTGGCTGAGAAGTTGGAACTCGAGTTCCAACTTCTCAGCCAACTC |
| FSP1 3’UTR | CTCGAGCACTTGCCAGGCGGGTGC | GTTTAAACAGAGGCCAGGCGGGAGAA |
